# Toward Increasing Engagement in Substance Use Data Collection: Development of the Substance Abuse Research Assistant App and Protocol for a Microrandomized Trial Using Adolescents and Emerging Adults

**DOI:** 10.2196/resprot.9850

**Published:** 2018-07-18

**Authors:** Mashfiqui Rabbi, Meredith Philyaw Kotov, Rebecca Cunningham, Erin E Bonar, Inbal Nahum-Shani, Predrag Klasnja, Maureen Walton, Susan Murphy

**Affiliations:** ^1^ Department of Statistics Harvard University Cambridge, MA United States; ^2^ Department of Psychiatry and Addiction Center University of Michigan Ann Arbor, MI United States; ^3^ Department of Emergency Medicine University of Michigan Medical School Ann Arbor, MI United States; ^4^ Injury Prevention Center University of Michigan Ann Arbor, MI United States; ^5^ School of Public Health University of Michigan Ann Arbor, MI United States; ^6^ Institute for Social Research University of Michigan Ann Arbor, MI United States; ^7^ School of Information University of Michigan Ann Arbor, MI United States

**Keywords:** engagement, microrandomized trial, just-in-time adaptive intervention

## Abstract

**Background:**

Substance use is an alarming public health issue associated with significant morbidity and mortality. Adolescents and emerging adults are at particularly high risk because substance use typically initiates and peaks during this developmental period. Mobile health apps are a promising data collection and intervention delivery tool for substance-using youth as most teens and young adults own a mobile phone. However, engagement with data collection for most mobile health applications is low, and often, large fractions of users stop providing data after a week of use.

**Objective:**

Substance Abuse Research Assistant (SARA) is a mobile application to increase or sustain engagement of substance data collection overtime. SARA provides a variety of engagement strategies to incentivize data collection: a virtual aquarium in the app grows with fish and aquatic resources; occasionally, funny or inspirational contents (eg, memes or text messages) are provided to generate positive emotions. We plan to assess the efficacy of SARA’s engagement strategies over time by conducting a micro-randomized trial, where the engagement strategies will be sequentially manipulated.

**Methods:**

We aim to recruit participants (aged 14-24 years), who report any binge drinking or marijuana use in the past month. Participants are instructed to use SARA for 1 month. During this period, participants are asked to complete one survey and two active tasks every day between 6 pm and midnight. Through the survey, we assess participants’ daily mood, stress levels, loneliness, and hopefulness, while through the active tasks, we measure reaction time and spatial memory. To incentivize and support the data collection, a variety of engagement strategies are used. First, predata collection strategies include the following: (1) at 4 pm, a push notification may be issued with an inspirational message from a contemporary celebrity; or (2) at 6 pm, a push notification may be issued reminding about data collection and incentives. Second, postdata collection strategies include various rewards such as points which can be used to grow a virtual aquarium with fishes and other treasures and modest monetary rewards (up to US $12; US $1 for each 3-day streak); also, participants may receive funny or inspirational content as memes or gifs or visualizations of prior data. During the study, the participants will be randomized every day to receive different engagement strategies. In the primary analysis, we will assess whether issuing 4 pm push-notifications or memes or gifs, respectively, increases self-reporting on the current or the following day.

**Results:**

The microrandomized trial started on August 21, 2017 and the trial ended on February 28, 2018. Seventy-three participants were recruited. Data analysis is currently underway.

**Conclusions:**

To the best of our knowledge, SARA is the first mobile phone app that systematically manipulates engagement strategies in order to identify the best sequence of strategies that keep participants engaged in data collection. Once the optimal strategies to collect data are identified, future versions of SARA will use this data to provide just-in-time adaptive interventions to reduce substance use among youth.

**Trial Registration:**

ClinicalTrials.gov NCT03255317; https://clinicaltrials.gov/show/NCT03255317 (Archived by WebCite at http://www.webcitation.org/70raGWV0e)

**Registered Report Identifier:**

RR1-10.2196/9850

## Introduction

Substance use remains a major public health issue [1,2] due to its associations with risky behaviors (eg, intoxicated driving, violence, risky sexual behaviors, etc) and short-term (eg, injury) and long-term health consequences (eg, development of substance use disorders) [3-6]. Substance use typically begins during early adolescence (typical age, 12-17 years), with emerging adults (typical age, 18-25 years) having the highest prevalence [5]. According to the National Survey on Drug Use and Health, 11.5% of adolescents reported alcohol use and 6.1% reported binge drinking (5 or more drinks) in the past month; 59.6% of emerging adults reported drinking and 37.7% reported binge drinking in the past month [5]. Marijuana is the most commonly used illicit drug in the United States, with 7.4% of adolescents and 19.6% of emerging adults reporting its use in the past month [5]. In addition, the legalization of medical and recreational use of marijuana in several states has paralleled the decreasing perceptions of risk [7-9]. This trend is concerning because marijuana use may affect the neuro-maturational development of the brain among youth [10-12], potentially compromising decision-making and inhibitory control functioning [10-13]. Finally, prescription pain medications are the next most commonly misused substance, with 7.4% of adolescents and 2.8% of emerging adults reporting use in the past month [5]. Importantly, the risk of overdose increases when binge drinking is combined with prescription opioids and sedatives [14].

Longitudinal panel studies show that, as youth develop, evolving interactions between individual, social, and community risk and protective factors can decrease or accelerate substance use trajectories [15-18]. However, there is a critical knowledge gap about the temporal processes that underlie substance use among youth (eg, youth have greater rates of binge drinking). Knowledge about these temporal processes can enable better understanding of the real-time antecedents and sequelae of substance use and can inform the development of just-in-time adaptive interventions (JITAIs) (eg, for craving, high-risk activity spaces, negative affect, and stress) [19-21]. Mobile phones provide a promising platform in this regard. Mobile phones are with us most of the time, which creates opportunities for frequent in situ data collection about the temporal processes underlying substance use. Among tech-savvy adolescents and emerging adults, mobile phones are also pervasive; 92% of teens (age, 13-17 years) go online daily, and 73% of them have a mobile phone, which is fairly balanced across racial groups, with 85% of African Americans, 71% of Caucasians, and 71% of Hispanics having a mobile phone [22].

However, sustaining self-reported data collection is challenging in mHealth [23,24]. Self-reporting rate is generally low for mHealth data collection apps [25], and the same is true for substance use apps. A recent review of mHealth apps for substance use (including short message service [SMS] text messaging and apps) concluded that engagement is a critical limitation, with most use declining quickly over brief periods of time (2 weeks to 3 months) [26], However, only few studies included adolescents or emerging adults [27]. The limited research on behavioral health apps among youth also found engagement challenging, potentially because youth can become habituated to apps [24] or due to competing demands from the frequent use of other apps, such as social media [22] or entertainment apps [28].

To date, there has been little work on increasing self-reporting rates of substance use data [29-31]. Sensor technologies can partially mitigate low self-reporting rates because sensors require no additional effort other than carrying the device [32,33]. However, detecting substance use by sensors is new and requires further validation. Bae et al [34] used mobile phone data to detect drinking episodes; SCRAM [35,36] and BACTrack Skyn [37] are wearable sensors that can continuously measure blood alcohol levels. However, such sensors do not exist for other substances, and important correlates of substance use such as stress level and mood cannot yet be reliably detected using sensors [38]. Therefore, self-reporting remains a valuable method to obtain substance use-related data. Previously, financial incentives were used, often along with frequent staff contact, to increase the rates of self-reporting [39-41]; however, both these strategies are prohibitively expensive for larger studies over longer periods of time. Other approaches are needed for engaging participants to self-report.

In this paper, we describe a mobile phone app, Substance Abuse Research Assistant (SARA), intended to enhance participant engagement in self-reporting. We also describe a micro-randomized trial (MRT) [42,43] design to rigorously test several *engagement strategies* we built into SARA. To our knowledge, the SARA study will be the first [44] to examine how the effect of engagement strategies may vary with both time and context (eg, negative affect, stress, loneliness). Our vision for SARA is an “engagement first” approach, where engagement strategies are employed to increase or sustain self-reporting over extended periods of time. An important goal of SARA is to reduce staff time and financial incentives using nonfinancial engagement strategies that are grounded in behavioral science theories (such as operant conditioning [45-48] and reciprocity [49-51]). In the future, we plan to use the collected data to trigger interventions aimed at reducing substance use. This paper provides a detailed description of SARA, including the theoretical foundation of SARA’s different engagement strategies, and describes the study design we used to test the efficacy of SARA’s engagement strategies over time.

## Methods

### Substance Abuse Research Assistant

**Figure 1:**
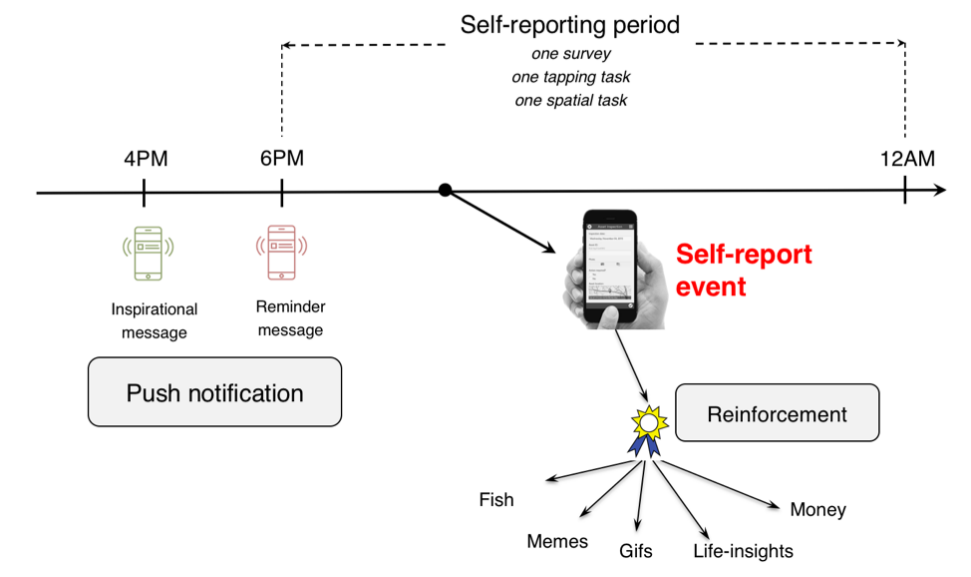
Daily timeline for engagement strategies in Substance Abuse Research Assistant (SARA). Push notifications are sent prior to data collection. Reinforcements are provided after data collection.

SARA is a mobile phone app aimed at increasing self-reporting of substance use data from adolescents and emerging adults. The app runs on both Android and iOS platforms. To collect data about correlates of substance use, every day, participants are prompted to complete a survey and 2 active tasks. SARA’s key innovation is the variety of engagement strategies it incorporates to incentivize and support this data collection. The base engagement strategy is a virtual aquarium, which starts empty, but as a participant provides more data, the fish population grows and treasures accumulate. In addition to the aquarium, other strategies such as push notifications with inspirational messages, memes, and informative visualizations of self-report data are used to enhance engagement with self-reporting. SARA consists of 2 modules: the data collection module and the engagement module (Figure 1).

#### Data Collection Module

SARA’s data collection module deals with procuring self-reported data. SARA currently supports 2 types of self-reported data collection. The first is provided by active tasks. Active tasks, which were first introduced in the Apple Research Kit [52], constitute intuitive user interactions that allow researchers to *objectively* measure reaction time, spatial memory, gait, problem-solving skills, etc. SARA’s first active task serves as a measure of spatial memory, a random sequence of 5 seashells lights up in a 2-dimensional grid of 9 seashells. Participants are then asked to repeat the sequence. The second active task is a tapping task in which participants tap 2 buttons alternately for 10 seconds. The number of completed taps gives a measure of reaction time. The reason for choosing the tapping and spatial tasks is that spatial memory and reaction time may vary based on substance use-related intoxication [53,54].

The second type of data collection is a daily survey in which participants report their daily feelings and activities (ie, stress level, mood, loneliness, hopefulness, amount of free time, and excitement) [55-58]. On Sundays, an extra set of questions assesses the past week’s frequency of substance use (alcohol, cannabis, and tobacco), motives and riskiness of use (for alcohol and marijuana), impulsivity, and intentions to avoid alcohol and marijuana use in the upcoming week [8,59-63]. Multimedia Appendix 1 contains the survey questions and the screenshots of the active tasks.

In the current version, participants self-report 1 survey and 2 active tasks (1 tapping task and 1 spatial task) every day between 6 pm and midnight. We selected this time window for the following reasons: (i) 6 pm to midnight provides a large enough time period to self-report, (ii) to capture a summary of most of the day using the survey in the evening, and (iii) to capture intoxication via active tasks since substances are typically used by youth in the evening [64].

#### Engagement Module

The engagement module of SARA contains several engagement strategies to increase self-report completion. These engagement strategies can be grouped as follows: (1) postdata collection rewards, (2) predata collection incentives, and (3) reminders to self-report. In addition, SARA contains other enhancements to support engagement, for example, occasional human support by SMS text messages or phone calls when participants temporarily disengage; a small amount of money, and an overall coherent user experience so that the app is easy to use, and the engagement strategies appear to be part of one app. Since SARA focuses on reducing financial incentives, we took several measures so that the engagement strategies are indeed effective for the target population.

First, we grounded the strategies in behavioral theories on influencing the intended action. Second, we followed a user-centered design process, where we conducted 3 focus groups (N=21, mean age 19.9 years) and a pilot study (N=17, mean age 21.2 years); a majority of the participants in these studies (67% in focus groups and 100% in the pilot study) reported binge drinking (5+ drinks at 1 occasion) or marijuana use in the past 3 months. We describe below the engagement strategies that are supported by theories and the user-centered design process.

##### Postdata Collection Rewards

Providing rewards after the successful completion of an intended behavior (eg, self-reporting in SARA) is a well-established method to shape future behavior [45,46]. Over the last few decades, the operant conditioning literature has extensively investigated how consequences shape behavior. According to the operant conditioning theory, the following are the 2 key ideas to influence the effectiveness of rewards: (1) Immediate contingent reward: rewards are more efficacious if they are given immediately and only after the intended behavior happens. Delaying rewards after the intended behavior or providing rewards after nonintended behaviors will make the reward less efficacious [47,48]. (2) Value of the reward: the rewards need to be valuable enough to trigger the intended behavior. One method to provide high enough value is to provide a variety of rewards; thus, even if one reward is less effective in a particular context, there is a higher likelihood that another reward can substitute for the less effective reward [65]. SARA employs these 2 ideas from operant conditioning as follows. First, ensuring immediacy and contingency was trivial; we provide the rewards immediately and only after self-reports. To ensure value, we have 2 types of rewards, (1) a growing virtual aquarium and (2) memes and life insights. We present below their design along with supporting evidence from prior work and user-centered design to show that the strategies can indeed engender reward value.

###### A Growing Virtual Aquarium

**Figure 2:**
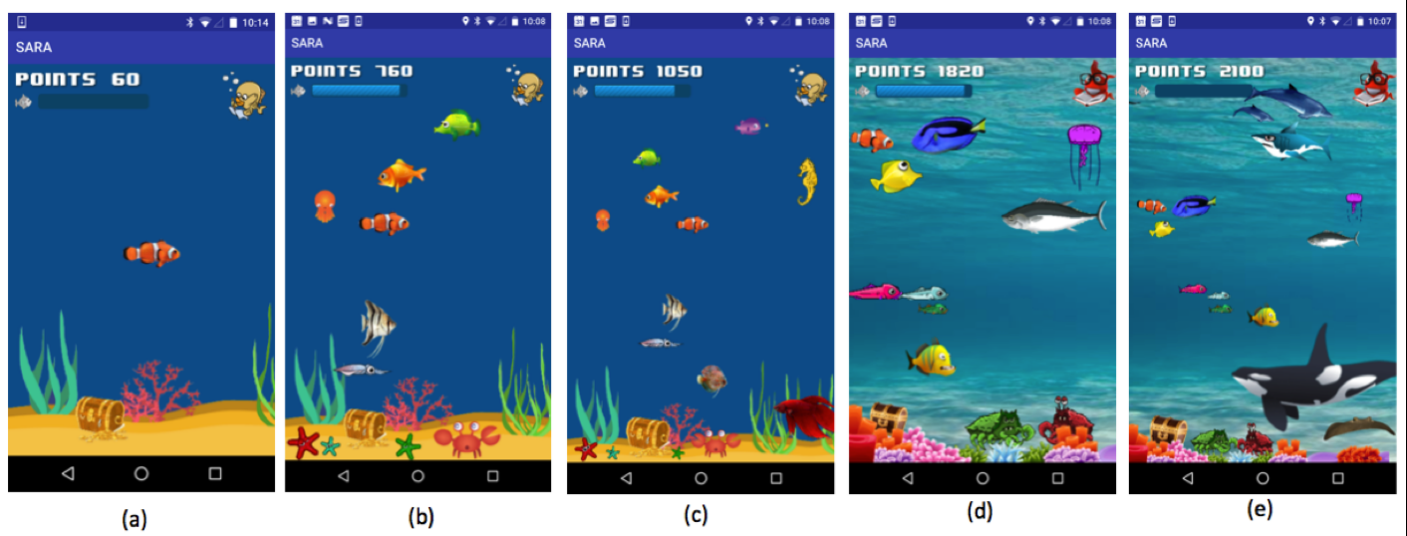
The evolution of Substance Abuse Research Assistant (SARA)’s aquarium environment as data is collected. Panels a, b, c, d, and e respectively show the state of aquarium if a participant self-reports for 1, 7, 14, 24, and 30 days.

In SARA, a virtual aquarium environment grows richer as more self-reports are completed (Figure 2). Every time participants finish either the survey or the 2 active tasks, they earn 30 points toward the aquarium. For the longer survey on Sundays, 50 extra points are rewarded. New fish are unlocked as specific numbers of points are accumulated. Multimedia Appendix 2 lists the fish in the SARA aquarium and the corresponding numbers of points that unlock them. SARA is set up so that 1 fish can be unlocked almost every day if both the survey and active tasks are completed. Every time a fish is unlocked, a fun fact about the fish is also given; for example, when a goldfish is unlocked, participants see the message “Do you know goldfish can recognize faces?” An exception to the 1-fish-a-day rule is made for the first 2 days of the study, when SARA provides 2 fish per day. Initially, these extra fish are given to quickly condition the participant to the fact that interesting fish are unlocked if they self-report [48]. SARA makes the aquarium environment more game-like by introducing levels; after 15 days of self-reporting, participants graduate from a fishbowl environment to a sea environment. Levels help prevent cluttering as more fish are unlocked, while increasing the participants’ interest. In addition, for streaks of self-reporting, participants can earn treasures such as pearls and gemstones. Multimedia Appendix 3 lists the different pearls and gemstones available in SARA and the corresponding self-reporting streaks that can unlock them.

The growing aquarium generates reward value based on the conceptualization of engagement as a “subjective experience” [44,47,48]. The aquarium is intended to generate positive subjective experience by creating enjoyment through collection of fish in a game-like environment. Rewarding self-reporting with points or fish also intends to promote positive subjective experiences by linking self-reporting behaviors with positive emotions (eg, joy and pride). Furthermore, once participants are initially engaged, the aquarium extends the positive experience by adding complexity through the aforementioned fun fish fact, levels, and treasures. Adding such complexity makes participants feel that their efforts are being reciprocated by the designers who have invested additional effort in creating new features and challenges; this sense of reciprocation may motivate participants to engage further [66].

Finally, an aquarium representation was chosen because aquariums have been used successfully to represent rewards in wellness apps in the past—notably in Fish n’ steps [67] and BeWell [68]. A recent commercial game known as “Abyssrium,” where a user has to grow an aquarium over time, has been downloaded more than 30 million times and has received the game of the year award in 2016 [69]. Participants in the user-centered design process also found the aquarium metaphor appealing (eg, focus group participants rated the SARA aquarium 3.9 stars out of 5 for use in a research study).

###### Memes, Gifs, and Life Insights

Although the aquarium is expected to promote engagement, it may lose its novelty over time. Therefore, SARA includes other post-data collection rewards in the form of memes or gifs and life insights. Once participants complete the daily survey part of the self-input, they may receive a meme or an animated gif. The meme or gif is intended to be either funny or inspirational. Memes and gifs are chosen because they can generate reward value by positive emotions and encouragement [44,47,48,70]. Furthermore, the nonjudgmental nature of included memes or gifs is consistent with other substance use interventions [70]. The memes and gifs in SARA were generated using Amazon’s Mechanical Turk and reviewed by undergraduate research assistants (RAs) who were of the same age as the target population. Participants in the user-centered design process for SARA also found the memes or gifs as acceptable forms of rewards (eg, on a scale of 1=strongly dislike to 5=strongly like memes or gifs as rewards for self-report, the average rating among a pilot study sample was 3.85).

In addition to memes or gifs, the participants may receive a life insight after they complete the active tasks portion of the data collection. Life insights are visualizations of self-reported data from the past. SARA’s life insights are trends of the various data collected using daily survey and active tasks over the past 7 days. SARA contains a life insight for each of the following data types: (1) daily stress, (2) amount of free time in the day, (3) degree of loneliness in the day, (4) level of fun on the day, (5) how new and exciting were the days, (6) tapping speed, and (7) seconds taken to finish the spatial task. Note that 1-5 are gathered from the daily survey and 6-7 are gathered from the active tasks (see Multimedia Appendix 4). Life insights can generate reward value because individuals strive to understand themselves and gain self-relevant knowledge [71-73]. People are frequently unclear about their personal abilities and they learn about themselves by attending to and seeking self-relevant information [71,72,74,75]. Consistent with this notion, previous work has demonstrated that people are interested in receiving feedback about their past self-reported experiences [76]; in fact, most health apps and wearables (eg, fitbit) use visualizations of past data to provide feedback to their users. Participants in the user-centered design process for SARA were also quite interested in seeing their data on life insights (eg, on a scale of 1=strongly dislike to 5=strongly like life insights as rewards for self-report, the average rating among a pilot study sample was 3.92).

##### Predata Collection Incentive

Sociopsychological perspectives [49-51] suggest that reciprocity, that is, returning a favor, is an innate human tendency. Drawing on these perspectives, SARA sometimes provides incentives before (ie, not conditional on) self-reporting to facilitate participant reciprocation via subsequent self-reporting. SARA may issue a youth-focused inspirational message as a push notification at 4 pm, 2 hour before the data collection period starts. We selected 4 pm because adolescents or emerging adults are likely to be out of school at that time and hence are likely to notice the notification. This time is also close enough to data collection time (6 pm) so that providing an incentive may facilitate participant reciprocation via survey or active task completion. To facilitate participant reciprocation, we provide inspirational messages. From the user-centered design process, we found inspirational quotes in the form of song lyrics and celebrity quotes, which might be appealing to youth. Please refer to Multimedia Appendix 5 for the list of quotes used in SARA. Once again, this repository of messages was assembled and filtered by the undergraduate RAs who were of the same age as our target population. In the pilot study, we asked participants how much they liked the inspirational quotes as an incentive for self-report (1=strongly dislike, 5=strongly like); the average rating was 3.3.

##### Reminder Notifications

Past research has demonstrated that reminders can increase engagement [44]. SARA thus provides a message at 6 pm to remind participants to report data. The reminder message is sometimes appended with additional content, such as “you are close to unlocking a new fish,” “you are close to finishing a streak and earning some money,” or “it only takes a minute to collect data in SARA.” The additional content tries to increase adherence by altering the perception of the value of self-reporting by reminding the participants of rewards that follow or that self-reporting does not require a lot of effort [77]. We selected 6 pm to send the reminder notification because it was the start time for the daily 6 pm to midnight self-reporting period discussed previously.

##### Other Enhancements to Support Engagement

SARA includes a few additional enhancements to support the abovementioned engagement strategies:

###### Financial Incentives and Human Support

One potential issue with the aquarium, memes, and life insights is that they are new rewards and participants may need time to perceive their full value. For example, unlocking fish and growing a virtual aquarium will be new to participants at the start of the study and they need to receive rewards several times before understanding what to expect. Therefore, participants may need additional sources of reinforcement that are rewarding right from the start [65]. Earlier work has demonstrated the utility of financial incentives [39,40] and human support [41] in promoting engagement. Participants in the user-centered design process for SARA also were very interested in financial incentives (eg, 95% of focus group participants reported that money bonuses would very much increase self-reporting in SARA). Hence, SARA includes relatively minimal financial incentives and human support to supplement its core engagement strategies; for every 3-day streak of self-reporting, that is, completing the survey and active tasks each day, participants can earn 1 dollar. For completing the longer weekly survey on Sunday, an extra 50 cents can be earned. For a 90% self-report completion rate, most participants can earn US $12 or less in a 30-day study (US $13 if self-report completion rate is 100%). Note that this is a fraction of what daily substance use studies normally pay for self-reporting (eg, US $1-4 dollars per day) [21,78].

In addition, if participants do not self-report, they receive SMS text messages from a study phone number. The first SMS text message is sent after 2 days of no self-reporting. If participants still do not self-report, a SMS text message is sent after 3 additional days of no self-reporting. The SMS text message follows a prespecified template (see Multimedia Appendix 6), which can be automated in future versions of SARA. After 7 days of no self-reporting, participants receive a phone call from a study team member. SMS text messaging and phone calls stop if participants neither respond nor self-report for 3 weeks.

###### User Experience

In SARA, we maintain a coherent user experience using an aquarium theme throughout the app. For example, in the spatial task, we use seashells to match the aquarium theme instead of flowers originally used by the Apple Research Kit [52,79]; after data collection, rather than only providing memes or life insights, we use animations consistent with the aquarium theme; divers swim into the aquarium and inform the participants that they earned a reward (eg, meme or life insight). Further attention-to-detail is provided to improve user experience; for example, Sunday’s survey contains 2 parts where participants answer a few questions about their day, followed by a few questions about the past week. Since Sunday’s survey is longer than other days, we include a fun question right after the daily questions to entertain and energize participants before asking them additional questions about the past week. This fun question is randomly selected from a set of 5 questions in Multimedia Appendix 7.

**Figure 3:**
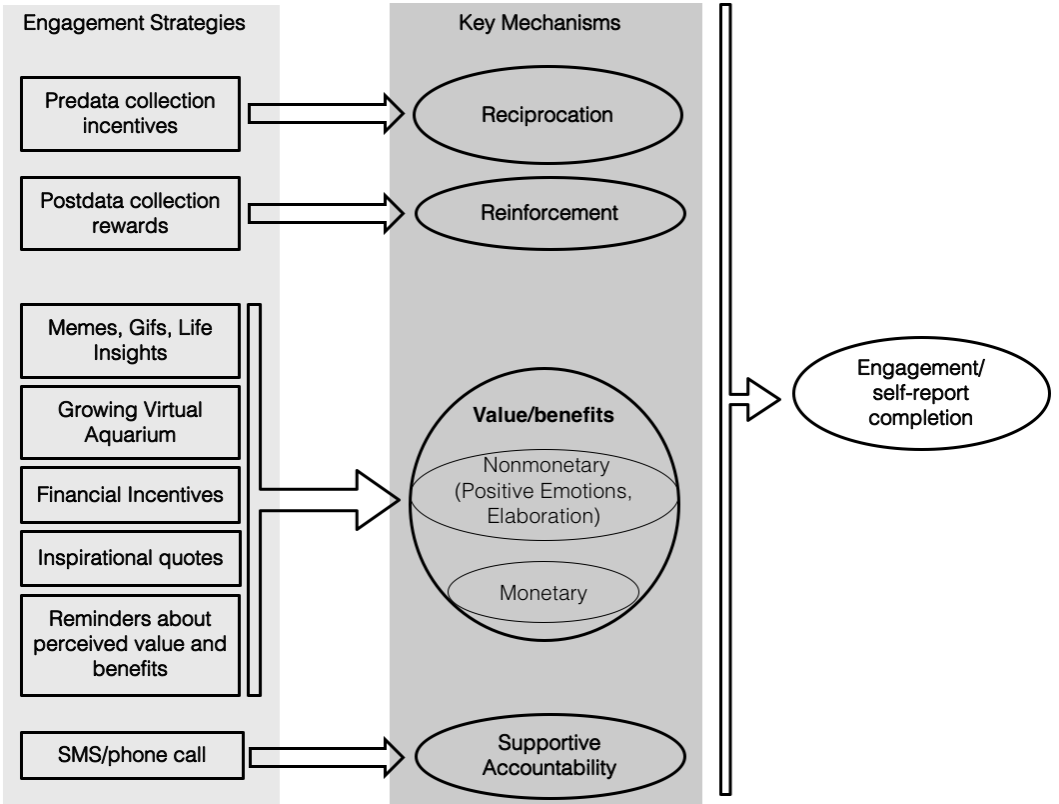
A conceptual diagram of how the different engagement strategies should affect self-report completion.

With this, we conclude the description of different engagement strategies in SARA. Figure 3 provides a summary of the engagement strategies and how these strategies affect various theoretical constructs to influence self-report completion in SARA.

#### Implementation Details

We used the cross-platform JavaScript framework Ionic to build the Android and iPhone versions of SARA. The aquarium part uses the Phaser 2D game library for the animations. All the self-reported data in SARA are encrypted to comply with the Health Insurance Portability and Accountability Act (HIPAA). All data are stored in Amazon S3, a HIPAA-compliant data storage service, and the communication between Amazon S3 and the mobile phone app is encrypted with RSA 2048 and AES-256 [80].

### Objective

In the previous section, we described several of SARA’s novel engagement strategies. Although SARA engagement strategies were designed to influence self-report completion among youth who use substances, the effectiveness of these strategies is unknown. Important questions include whether different SARA engagement strategies lead to higher self-report completion and how their effectiveness is moderated by context (eg, negative affect, stress, loneliness, etc). Ineffective engagement strategies may aggravate participants, and participants may habituate even to an initially effective engagement strategy [81,82], resulting in a decrease, over time, in the effectiveness of the strategy. Similar drawbacks might occur if an engagement strategy is used in a context in which it is ineffective; indeed, the current context of each participant may influence the effectiveness of an engagement strategy [77]. One way to gain insight into these questions is by experimentally manipulating the engagement strategies over time. In other words, the manipulation can inform the development of a policy for adaptive engagement strategy delivery to keep participants engaged in self-reporting. Recently, MRTs have been proposed as a method to develop JITAIs for mHealth [42,43]. In SARA, the intervention is composed of the engagement strategies, such as giving a meme or issuing an inspirational message. In MRTs, study participants are randomized sequentially, often multiple times a day, to receive different intervention components. Several key elements in MRTs are decision points and proximal outcomes. *Decision points* refer to the time points when participants are randomized. In SARA, there is a 4 pm decision point at which a participant is randomized to receive an inspirational message via push notification or receive nothing. Once a participant is randomized at a decision point, the *outcome* of using an engagement strategy can be measured *proximally*. A proximal measure for the 4 pm inspirational message is whether or not the participant self-reports later on the same day. For more details on MRTs, we refer the readers to [42,43,83]. In the following, we describe the MRT protocol for SARA.

### Study Protocol

We plan to run a 30-day MRT. Prior to the study, potential participants are screened, and eligible participants complete an in-person intake session with a study recruiter. Within the 30-day study, each participant is randomized at each of 4 decision points per day. After the 30 days, each participant takes part in a follow-up phone interview and provides feedback on using SARA for a month. The details of the study are as follows:

#### Study Setting and Eligibility

The 30-day MRT is conducted at the University of Michigan. Study participants are recruited from the University of Michigan Hospital Pediatric and Adult Emergency Departments. The study is approved by the Institutional Review Board of the University of Michigan Health System (HUM00121553) and is registered at ClinicalTrials.gov (NCT03255317). Patients are eligible for screening if they are aged between 14 and 24 years, understand English, are medically stable, are able to provide informed consent or assent (eg, not cognitively impaired or intoxicated), and are accompanied by a parent or guardian (for patients aged between 14 and 17 years). Individuals are eligible if they 1) have an Android or an iPhone mobile phone on which the app can be downloaded and 2) screen positive for past-month binge drinking (≥4 drinks for females and ≥5 drinks for males, on at least 1 occasion) or past-month cannabis use without a medical marijuana card.

#### Baseline Procedure

At the University of Michigan Emergency Department, recruiters monitor incoming admissions and identify patients in the target age range who do not meet the screening exclusion criteria (eg, presentation for sexual assault, droplet precautions, active vomiting, roomed in critical care). After completing an online consent or assent, participants self-administer a screening survey on a tablet, which contains questions regarding their demographics [84,85], health behaviors such as alcohol and marijuana use [8] and sleep habits [86], cell phone capabilities, and social media use. The screening survey takes approximately 8 min to complete, and participants receive a small gift valued at US $1.00 (eg, headphones, water bottle, mobile phone armband) for completing it. Those who report past-month binge drinking or marijuana use and have access to their mobile phone while in the Emergency Department provide written consent or assent and self-administer an approximately 20-min baseline survey with items about (1) substance use frequency, consequences, overdose, and driving under the influence [86-88]; (2) violence involvement, injury, and risky sex behaviors [6,89-92]; (3) coping and mindfulness [93,94]; (4) social influences [95-97]; and (5) motivation or self-efficacy to reduce their alcohol and marijuana use [98,99].

Then, the recruiters collect contact information from the participants. Recruiters ensure that participants install SARA on their phone during the intake since in the pilot study prior to this trial, we have found that many potential participants did not install the app after they left the intake session. Basic instructions on how to use the app are provided during intake, followed by motivational statements regarding participant’s stated barriers to using SARA regularly. Participants receive US $20 cash for completing their intake visit.

#### The 30-Day Microrandomized Trial

Following the intake session, participants start the 30-day MRT with SARA. Each day, participants are requested to complete 1 survey and 2 active tasks between 6 pm and midnight. Participants are randomized at the following 4 decision points each day:

1. *4 pm reciprocity notification*: Every day at 4 pm, with a probability of .5, participants are randomized to either receive a push notification with an inspirational message—a song lyric or a quote from a contemporary celebrity—or to not receive anything.
2. *6 pm push reminder notification*: Every day at 6 pm, a reminder notification is issued. The notification may be one of the following 2 types, randomized at .5 probability:
  - A simple reminder to complete a survey and 2 active tasks
  - A reminder to complete a survey and 2 active tasks, along with an additional persuasive message. The additional persuasive message may be one of the following 3 messages:
    - “Do you know it only takes a minute to fill out the survey and active tasks?”
    - “Do you know you can earn money if you complete a 3-day streak?”
    - “You are close to unlocking the next fish for the aquarium.”
3. *Reinforcement after survey completion*: If the survey is completed, participants are randomized at .5 probability to receive or not receive either a meme or a gif as a positive reinforcement. If a reinforcement is delivered, there is a .5 probability to receive a meme and a .5 probability to receive a gif.
4. *Reinforcement after active task completion*: If active tasks are completed, participants are randomized at .5 probability to receive or not receive a life insight as a positive reinforcement.

Note that not all the engagement strategies in SARA are randomized. The randomizations in 1-4 mentioned above are motivated by scientific questions concerning the abovementioned engagement strategies in SARA. Specifically, we randomize the push notifications because they can interrupt the participant and are intrusive, thus potentially reducing engagement. The other engagement strategies are not push notifications, so there is low risk of interruption; for example, the participant decides whether to open the app, complete self-reports, and receive the rewards. We randomize the memes and life insights because of the scientific question of how funny and informative contents respectively can influence self-report completion over time [44].

##### Exit Procedure

Participants are contacted by telephone to complete a 1-month follow-up interview, which includes the identical measures as the screen or baseline or daily survey or Sunday’s survey, as well as the following 2 new threads of questions: (1) a 30-day Timeline Followback calendar, which captures past-month alcohol and marijuana consumption [100] and (2) Likert-type and open-ended questions to capture user experience of SARA [101]. For completing the follow-up interview, participants receive a US $30 electronic gift card of their choice (eg, Amazon, Starbucks, etc).

### Analysis Plan

In this section, we describe the analysis plan to evaluate SARA. A more detailed version of the analysis plan can be found in a Center for Open Science document [102] (submitted on October 23, 2017).

#### Outcome Measures

The proximal outcomes of the randomizations are whether participants completed the survey and active tasks. For the inspirational message notifications at 4 pm and the reminder notifications at 6 pm, the proximal outcome is whether the survey or the active tasks are completed in the evening on the same day. For the 2 reinforcement interventions, the proximal outcome is whether the survey or the active tasks, respectively, are completed on the following day. Note that our engagement strategies are primarily designed to encourage self-reporting on the same or next day. Although the strategies may have longer term effects as well [81,82], we are interested in their effects on the same day or the next day; if these effects are consistently higher, then longer term adherence will be higher too. In addition, poststudy open-ended feedback from the participants will be analyzed to refine future versions of SARA.

#### Primary and Secondary Analyses

Our primary analyses will concern the following 2 hypotheses:

1. Providing the 4 pm reciprocity notification will yield a higher rate of full completion of the survey or active task on the same day than providing no intervention (*P*<.025).
2. Among individuals who complete the survey, providing a postsurvey-completion meme or gif will yield a higher rate of completion of the survey or active task on the next day than not providing meme or gif reinforcement after survey completion (*P*<.025).

We selected these hypotheses as primary since our team found these hypotheses to be the most interesting scientifically. The 4 pm randomization is designed to address the question of whether a notification intended to facilitate reciprocation (by providing an inspirational message before self-report time) is useful, and the randomization of participants upon survey completion is designed to investigate whether providing a postdata collection reward increases data collection. Furthermore, we conjecture that these hypotheses have the greatest potential to be supported. In particular, as the 4 pm notification is proximal in time to the data collection (2 hours prior), there are fewer extraneous distracting circumstances that can occur during this short time window that would reduce the intervention effect. Both hypotheses also consider a contrast between an active agent (ie, the 4 pm notification or the meme) versus nothing. Since these 2 hypotheses are primary, we divide the standard *P* value of .05 by 2.

Our secondary analyses will concern the following 2 hypotheses:

1. The 6 pm reminder notification with an extra persuasive message will yield a higher rate of full completion of the survey or the active task on the same day than not providing the extra persuasive message.
2. Among individuals who complete the active tasks, offering a postactive task-completion life insight will yield a higher rate of the full completion of the survey or active task on the next day than not offering a life insight after active task completion.

We consider the first hypothesis as secondary because the randomization for the 6 pm reminder is between 2 active agents (reminder with vs without a persuasive message). This implies that the additional effect of the persuasive messages may be small; thus, this hypothesis has a more exploratory nature. We consider the second hypothesis as secondary because data must accumulate before the visualizations in life insights become interesting. In addition, this hypothesis has a more exploratory nature because our life insights are only visualizations of past data; in the future, we aim for potentially more potent life insights using prediction tools [103,104].

For both primary and secondary analyses, we will control for the following 3 covariates to reduce variance in the outcome of self-input completion: (1) whether the survey or the active tasks were fully completed on the previous day, (2) whether SMS text messages or phone calls were made in the last 24 hours, and (3) whether the app was opened in the prior 72 hours outside when a survey or active task was completed. We will not include other baseline variables such as gender and age as covariates in the primary analyses because inclusion of a covariate uses up degrees of freedom. We also anticipate that the *within-person* covariate “whether the survey or active tasks were fully completed on the previous day” will capture some of the variance due to baseline gender or age. We will also not include time as a covariate in the primary analyses for the abovementioned reasons as well. Note that the statistical methods [83] will adjust the standard errors of the estimated effects to account for within-person correlation across time in the outcome. For more details about our primary analysis plan, please refer to the Center for Open Science document [102].

#### Exploratory Analyses

We plan to run exploratory analyses to examine how the effectiveness of engagement strategies changes over time (we conjecture that the effectiveness will decrease). We will also run additional exploratory analyses to assess effect moderation. We will examine how the effect of engagement strategies is moderated by gender, weekdays versus weekends, and whether the day is Sunday versus other days of the week (we expect that the completion rate for the longer Sunday’s surveys may be lower than that for other days). We initially planned to assess age and phone type (Android or iPhone) as moderators; however, we did not include these variables because recruitment thus far indicates that very few participants in the study have Android phones or are below the age of 18 years.

#### Sample Size

We started recruiting for the study on August 21, 2017. Recruitment concluded on February 28, 2018. We recruited 73 participants for the study.

#### Statistical Analyses

In mHealth, it is common to collect time-varying measures of the participants’ context (such as stress, mood, and loneliness from the self-report assessments). The provision of engagement strategies is time-varying as well; that is, at each decision point, participants can receive different options of the engagement strategy. A key statistical issue is that covariates (measures of the participant’s context) at a time point can be affected by past engagement strategies. For such a setting, Boruvka et al [83] proposed a method to estimate the causal effects of interventions on continuous outcomes. However, in SARA, we are dealing with a binary outcome (whether participants self-reported or not). Multimedia Appendix 8 contains details of a method that we have developed, which extends the work of Boruvka et al to binary outcomes. We will use RStudio 1.1.453 to run the statistical analysis.

The open-ended qualitative data from exit interviews will be coded using thematic analysis [105]. The qualitative analysis will be performed using NVivo 11.

#### Missing Data

Since our outcome is adherence to self-report completion, not completing self-reports is not missing data for our study. However, missingness can happen in the study if participants uninstall the app in the middle of the study. To account for such missingness, we will conduct the following 3 versions of the primary analysis: (1) only include participants who had the app installed for 30 days, (2) include all participants and include only days when the app was installed, and (3) include all participants and data for both installed and uninstalled days; for the uninstalled days, the 4 pm and 6 pm notifications will be imputed, and the outcome will be considered as “self-report noncompletion.” More details on how missingness will be accommodated can be found in the Center of Open Science document [102].

## Results

We started recruiting for the study on August 21, 2017. Recruitment concluded on February 28, 2018. We recruited 73 participants for the study. Data analysis is currently underway.

## Discussion

### Future Work

To the best of our knowledge, this study is the first MRT to systematically explore the efficacy of different engagement strategies on increasing self-reporting of substance use data among adolescents and emerging adults. The results of this trial will answer how different engagement strategies affect self-reporting and to what degree (ie, effect size). We will also learn whether the effectiveness of the strategies varies over time. The qualitative data from the exit interviews will help us to further triangulate and understand the findings of the quantitative analysis. Moreover, the exit interviews’ open-ended feedback about the app will help us further fine-tune the app.

For future studies, the analysis of the collected data can be used to initialize machine learning algorithms with the goal of providing engagement strategies in contexts (eg, stress level, loneliness, location, and weekend or weekdays), and at times, they are most effective. In particular, we will use the resulting data to train an initial policy for reinforcement learning algorithms such as “contextual bandits” [106]. As data accumulate on a participant, these algorithms increase the chance of providing the engagement strategy option that is most effective in a particular context and decrease the chance of providing an engagement strategy option that is less effective [107].

Another important direction of future work is the development of therapeutic interventions to prevent substance misuse. The daily surveys and active tasks will provide both subjective and objective data on substance use and related factors (eg, mood) over time. The Sunday’s survey will uncover the days when substance use events happened; we will use these data as labels and the daily survey (ie, stress, emotion, or loneliness) or active tasks (ie, spatial memory, reaction time) as features to create machine learning models of impending substance use events. Therapeutic interventions will be provided, with high probability, at the time of impending substance use (or when participants are likely to engage in intervention content). Future versions of SARA will integrate these JITAIs to reduce substance misuse. Note that data-driven JITAIs to reduce substance use require maintenance of sufficient engagement of self-input completion.

Finally, one more future direction is to include sensor data collection from phones and wearables. Sensor data can be useful in multiple ways for SARA, such as (1) reducing self-input burden with predictions, for example, loneliness can be measured by inferring social interactions from the phone [33]; reducing self-input burden may increase engagement [77] and (2) risky times of substance misuse can be preanticipated from sensors; we expect that substance use events co-occur with certain behavioral markers that can be captured using sensors, for example, when participants are close to liquor stores or texted friends with whom they previously engaged in substance misuse [34].

### Limitations

Few limitations of this study are as follows: first, this study is not designed to confirm that the SARA is more effective than other apps in collecting substance use-associated data. This trial is only designed to optimize the further development of SARA. We believe that this is a necessary first step since the science of engagement in mHealth is currently in its infancy. This MRT will inform the selection and adaptation of engagement strategies, as well as the development of future versions of SARA, which can be used as an experimental arm in randomized trials.

Second, the current version of SARA is limited because the design primarily focused on increasing willingness, that is, to the extent a participant is motivated to engage in self-reporting. Prior literature suggests 2 other methods to influence engagement [44,77,108-111]: (1) need, namely an individual’s recognition that there is a discrepancy between his or her present state and a preferred future state, and (2) ability, namely the extent to which the individual has the knowledge, experience, skills, and capacity to engage in data collection. Although SARA is not currently designed to experiment with ability or need, SARA may influence them indirectly; for example, the reminder prompts before data collection can address forgetfulness and enhances the ability of participants to collect data. Furthermore, SARA sets clear goals, monitors engagement with aquarium progression, and offers timely feedback [112]. Thus, if participants become engaged with the aquarium or other incentives, they may recognize the need to engage.

A third limitation is the limited funding of the SARA mobile app, which is novel and exploratory in nature. Limited funding constrained our sample size. We also could not implement several features that were requested during user-centered design process; for example, better aesthetics of the fish, interactivity such as touching and interacting with fish, or personalization of such customizable background of the aquarium. In the future, we will use the results of this pilot study and apply for grants that can support larger studies and more resources for app development.

Finally, the study lacks therapeutic interventions to improve substance use outcomes. However, the engagement-only approach provides naturalistic data on substance use and related factors at the daily level, allowing us to study in-the-moment precedents and sequelae of substance use among adolescents and young adults. A better understanding of the in-the-moment precedents and sequelae of substance use is necessary to shape future therapeutic interventions that can be integrated into SARA.

## Appendix

Multimedia Appendix 1 Survey questions and active tasks

Multimedia Appendix 2 Fishes and points

Multimedia Appendix 3 Gems and pearls

Multimedia Appendix 4 Screenshots of life-insights

Multimedia Appendix 5 Inspirational quotes.

Multimedia Appendix 6 Protocol for texting and phone calls.

Multimedia Appendix 7 Fun questions in the weekly survey

Multimedia Appendix 8 Analysis steps to assess causal effects

## Abbreviations

EMA: ecological momentary assessment
HIPAA: Health Insurance Portability and Accountability Act
JITAIs: just-in-time adaptive interventions
MACQ: Marijuana Consequences Questionnaire
MRT: microrandomized trial
RAs: research assistants
SARA: Substance Abuse Research Assistant
