# Supplementary material for: Toward Increasing Engagement in Substance Use Data Collection: Development of the Substance Abuse Research Assistant App and Protocol for a Microrandomized Trial Using Adolescents and Emerging Adults
Source: JMIR Res Protoc. 2018 Jul 18;7(7):e166. doi: 10.2196/resprot.9850 (PMC6070723; doi:10.2196/resprot.9850)
Supplement: Multimedia Appendix 1 [file resprot_v7i7e166_app1.pdf]

Daily survey

These questions are about how you’re feeling today.

1. How stressed are you today?

0	1	2	3	4
Not at all		Somewhat		A lot

2.

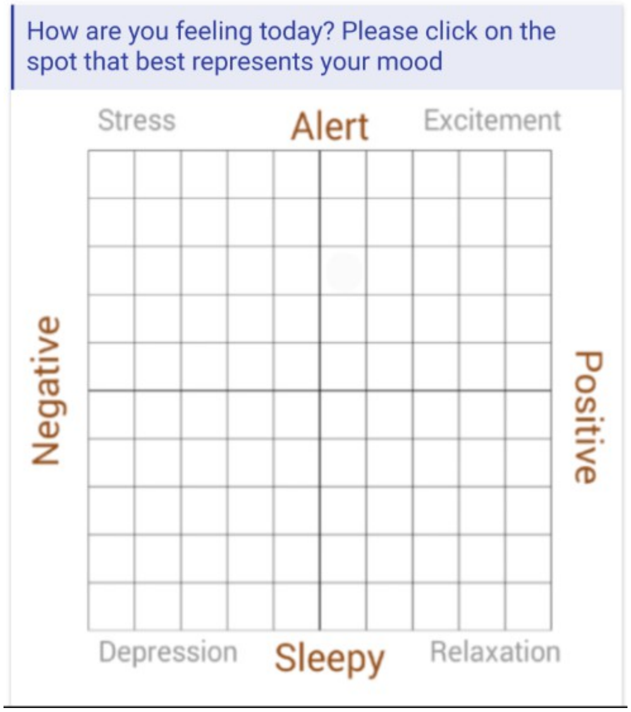

3. How much free time have you had today?
    - Less than 1 hour
    - Between 1 and 2 hours
    - Between 2 and 3 hours
    - Between 3 and 4 hours
    - Between 4 and 5 hours
    - Between 5 and 6 hours
    - Between 6 and 7 hours
    - Between 7 and 8 hours
    - Between 8 and 9 hours
    - Between 9 and 10 hours
    - Between 10 and 11 hours
    - Between 11 and 12 hours
    - Between 12 and 13 hours
    - Between 13 and 14 hours
    - Between 14 and 15 hours

Between 15 and 16 hours
16 or more hours

4. How much fun have you had today?

0	1	2	3	4
Not at all		Some		A lot

5. How lonely or left out have you felt today?

0	1	2	3	4
Not at all		Somewhat		Very

6. How new and exciting has your day been?

0	1	2	3	4
Not at all		Somewhat		Very

7. Do you expect good things will happen to you tomorrow?

0	1	2	3	4
Not at all		Somewhat		Very Much

Active tasks

Tapping task

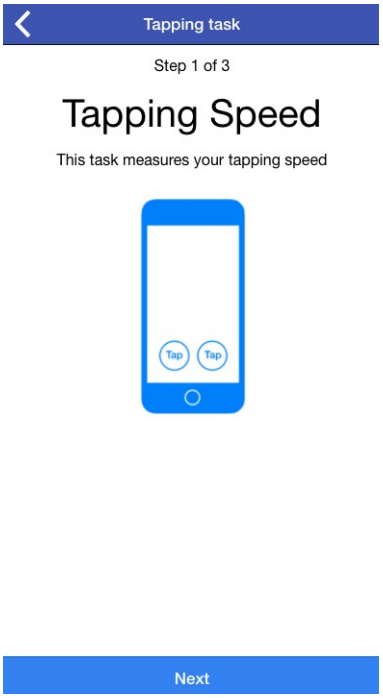

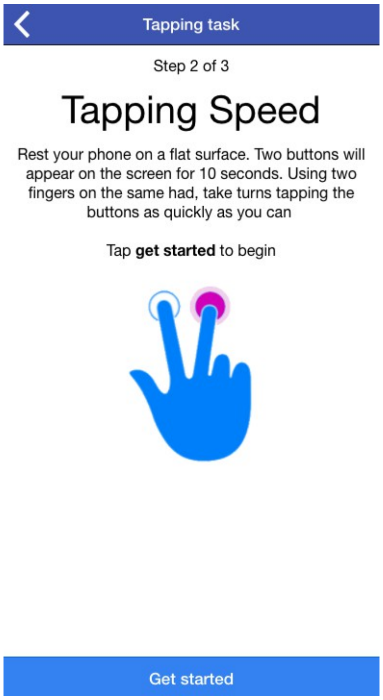

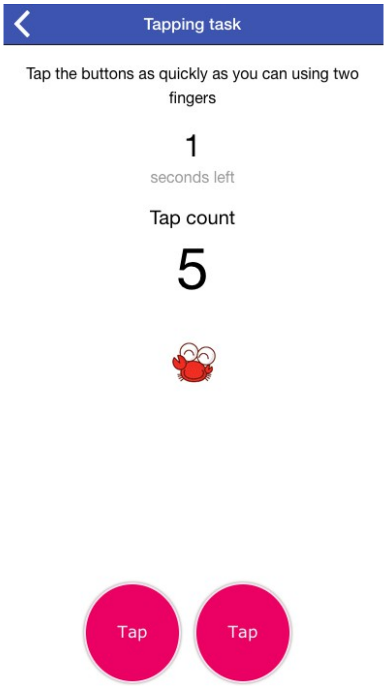

Spatial task

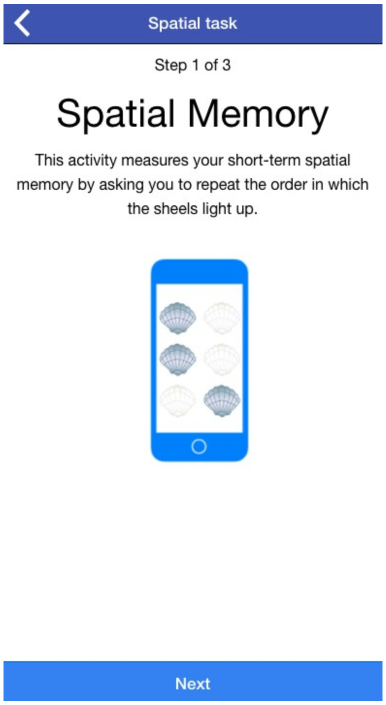

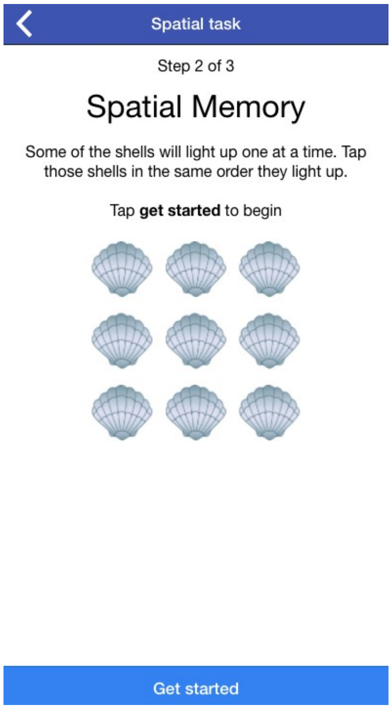

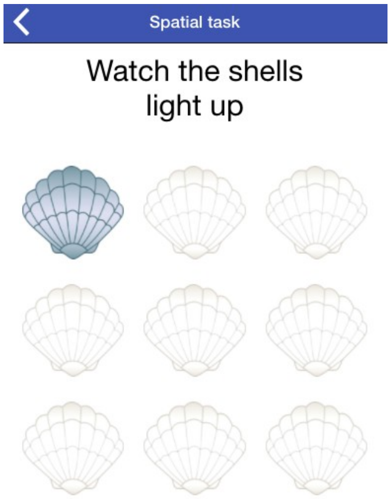

Weekly survey

This past week, did you…

1. Do or say things without thinking?
   - Rarely/Never
   - Occasionally
   - Often
   - Almost Always/Always

2. Plan tasks carefully?
   - Rarely/Never
   - Occasionally
   - Often
   - Almost Always/Always

3. Concentrate easily?
   - Rarely/Never
   - Occasionally
   - Often
   - Almost Always/Always

4. How frequently have you smoked cigarettes <u>this week</u>?

Not at all
Less than one cigarette per day
1-5 cigarettes per day
About ½ pack per day
About 1 pack per day
About 1 ½ packs per day
Two packs or more per day

5. Electronic vaporizers make a mist that is inhaled and have the feel of cigarette smoking. Examples include e-cigarettes and e-pens. How many days <u>this week</u> did you use an electronic vaporizer, such as an e-cigarette, to inhale a *nicotine* mist?

0
1
2
3
4
5
6 or more days

6. This week, how many drinks did you have on…

Sunday?
Monday?
Tuesday?
Wednesday?
Thursday?
Friday?
Saturday?

Response options for each day should be:

0
1
2
3
4
5
6
7
8
9
10
11
12
13
14
15
16
17
18
19
20+

If participant reports 1+ drinks in the past week show Q7a
If participant reports 0 drinks in the past week show Q7b

7a. Pick the top reason(s) why you drank this week:
To enjoy the feeling
To avoid feeling left out
To feel less depressed, anxious or angry
To make social gatherings more fun
Other (please specify: ____)

7b. Pick the top reason(s) why you didn't drink this week:
Didn't want to
No chance/time
Out of alcohol
Trying to cut back
More important things to do
Cost
Other (please specify: ____)

[Each week, participants will receive one question from the group of questions numbered 8a, 8b, 8c, 8d or 8e]

8a. What do you dislike, for no good reason?
Mayonnaise
Dryer lint
People who brag on Facebook
The word "moist"
Name brand addicts
Country music
Reality TV
None, I like all of these things

8b. If you were stranded on a desert island, what one food, what would you bring?
Ramen noodles
Cookie dough ice cream
Nacho cheese Dorito's
Power bars
Pop Rocks
French fries
Forget food, I'm bringing coffee
Gross. Who would take this stuff to a desert island?

8c. What movie best describes your life?
Breakfast Club
A Nightmare on Elm Street
Love Actually
Clueless
Kickass
Diary of a Wimpy Kid
Barely Legal

None of these; my life deserves its own movie

8d. If you were an animal, what would you be?

- Dinosaur
- Alligator
- Unicorn
- Cockroach
- Wallaby
- Dragon
- Charizard
- I’m way cooler than any of these animals

8e. If you could hang out with a movie character for one day, who would you pick?

- Harry Potter
- Ironman
- Jack Sparrow from Pirates of the Caribbean
- Fat Amy from Pitch Perfect
- Katniss Everdeen from Hunger Games
- Napoleon Dynamite
- Elle Woods from Legally Blonde
- Um, none of them. These characters are lame

9. This week, how much marijuana did you use on…

- Sunday?
- Monday?
- Tuesday?
- Wednesday?
- Thursday?
- Friday?
- Saturday?

Response options for each day should be:

- 0g
- 0.25g (1/2 joint)
- 0.5g (1 joint)
- 1g (2 joints)
- 1.5g (3 joints)
- 2g (4 joints)
- 2.5g+ (5+ joints)
- Not sure

If participant reports any marijuana in the past week show 10a only
If participant reports no marijuana in the past week show 10b only

10a. Pick the top reason(s) why you used marijuana this week:

- To enjoy the feeling
- To avoid feeling left out
- To expand your awareness
- To feel less depressed, anxious or angry
- To make social gatherings more fun
- Other (please specify:_______________)

10b. Pick the top reason(s) why you didn't use marijuana this week:

Didn't want to
No chance/time
Out of weed
Trying to cut back
More important things to do
Cost
Other (please specify:_______________)

These next two questions are about what you think you will do <u>next week.</u>

11. In the next week, how likely are you to avoid using marijuana if you have the chance?

0	1	2	3	4
Not at all				Very

12. In the next week, how likely are you to avoid drinking alcohol if you have the chance?

0	1	2	3	4
Not at all				Very

How much do you think people your age risk harming themselves (physically or in other ways) if they…

13. Use marijuana regularly?

Not at all, Slightly, Moderately, Very, Extremely

14. Drink alcohol regularly?

Not at all, Slightly, Moderately, Very, E
