# Supplementary material for: Toward Increasing Engagement in Substance Use Data Collection: Development of the Substance Abuse Research Assistant App and Protocol for a Microrandomized Trial Using Adolescents and Emerging Adults
Source: JMIR Res Protoc. 2018 Jul 18;7(7):e166. doi: 10.2196/resprot.9850 (PMC6070723; doi:10.2196/resprot.9850)
Supplement: Multimedia Appendix 2 [file resprot_v7i7e166_app2.pdf]

Daily survey: 30 points
2 active tasks: 30 points
Extra: 50 points for Sunday's survey

Fish bowl environment

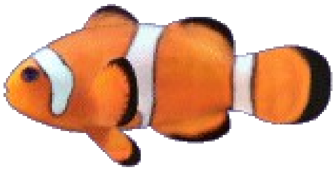	clown fish<br>Points: 30<br>Day 1
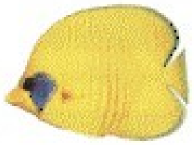	Butterfly fish<br>Points: 60<br>Day 1
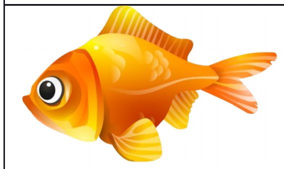	Gold fish<br>Points: 90<br>Day 2
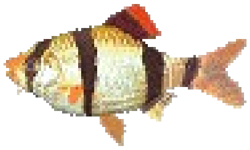	Tiger barb<br>Points: 120<br>Day 2
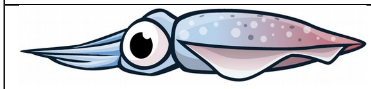	Squid<br>Points: 180<br>Day 3
GAP	GAP
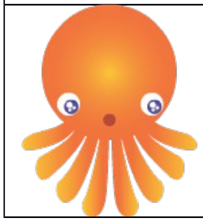	Octopus<br>Day 5:<br>Points 300

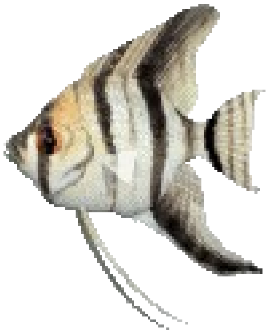	Angel fish, give 2 angel fish.<br>Points: 360<br>Day 6
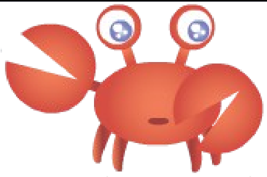	Crab<br>Points: 470<br>Day 7
GAP	GAP (need 120 points to get next fish)
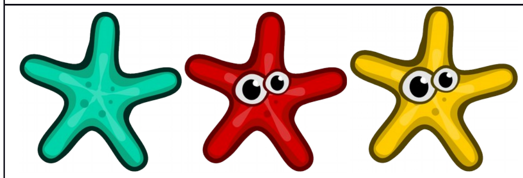 35	Star fish<br>Points: 590<br>Day 9
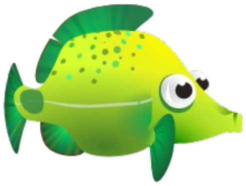	Green fish (60 pts after getting crab)<br>Points: 650<br>Day 10
GAP	GAP (need 120 points to get next fish)
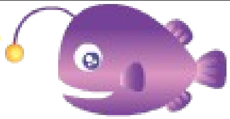	Electric fish<br>Points: 770<br>Day 12
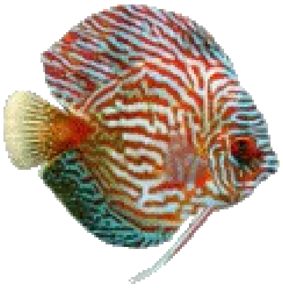	Discus fish<br>Points: 830<br>Day 13

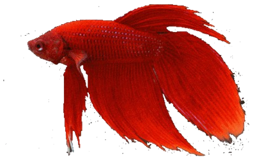	Red Betta fish<br>Points: 940, weekly survey<br>Day 14
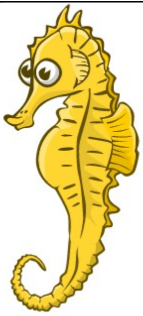	Sea horse, give 3 sea horse.<br>Sea horse, fish<br>Points: 1000<br>Day 15

Level 2: The sea

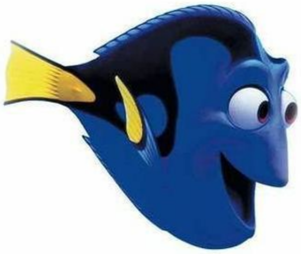 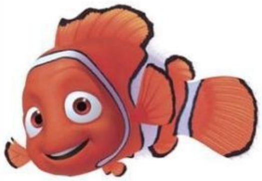	Blue tang fish and clown fish<br>Points: 60<br>Day 1
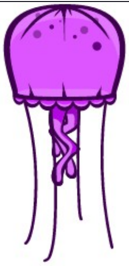	Jelly fish<br>Points: 120<br>Day 2
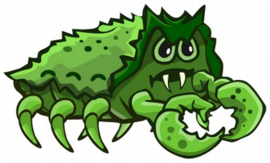 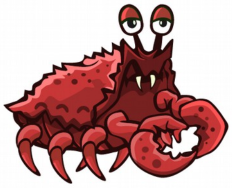	Sea crabs<br>Points: 180<br>Day 3

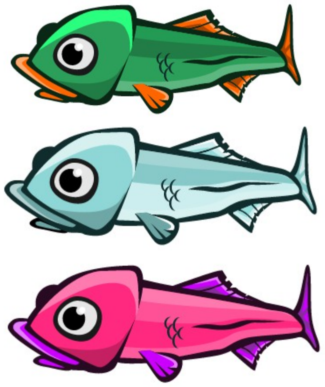	Three anchovies<br>Points: 240<br>Day 4
GAP	GAP
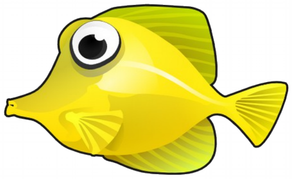 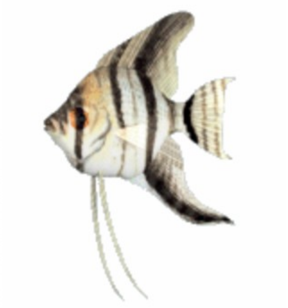	Bubbles<br>Angel fish<br>Points: 360<br>Day 6<br><br>Points: 470, 2nd fish, weekly survey.<br>Day 7
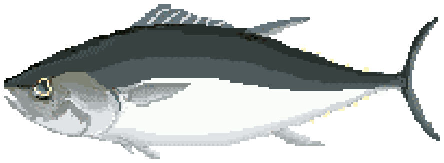	Salmon<br>Points: 530<br>Day 8
GAP	GAP
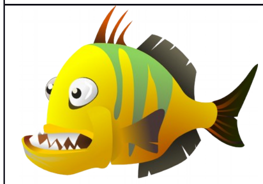	Deep undersea fish<br>Points: 650<br>Day 10
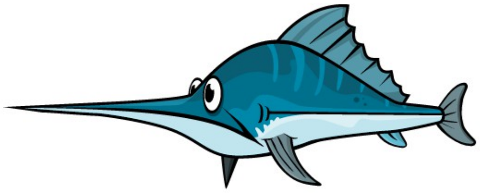	Give the sword fish<br><br>Points: 710<br>Day 11
GAP	GAP

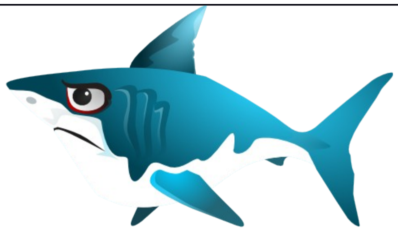	Shark<br>Points: 830<br>Day 13
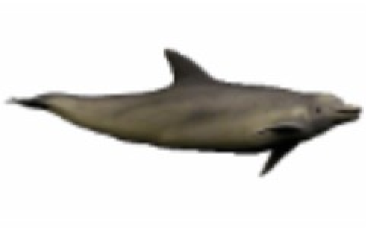	Dolphin<br>Points: 940<br>Day 14, weekly survey
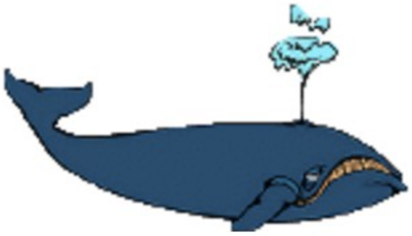	Whale<br>Points: 1000<br>Day 15
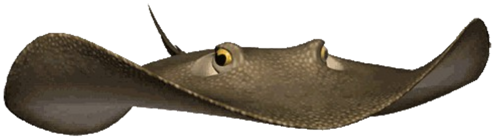	Kite fish<br>Points: 1060<br>Day 15
