# Supplementary material for: Toward Increasing Engagement in Substance Use Data Collection: Development of the Substance Abuse Research Assistant App and Protocol for a Microrandomized Trial Using Adolescents and Emerging Adults
Source: JMIR Res Protoc. 2018 Jul 18;7(7):e166. doi: 10.2196/resprot.9850 (PMC6070723; doi:10.2196/resprot.9850)
Supplement: Multimedia Appendix 3 [file resprot_v7i7e166_app3.pdf]

Rewards for daily survey streaks

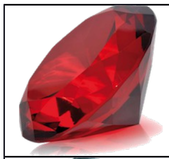	**Ruby stone**<br>For each 3 day streak<br>of daily surveys
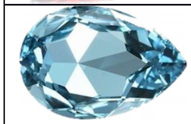	**Aquamarine**<br>For each 6 day streak<br>of daily surveys
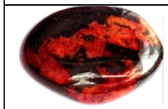	**Bloodstone**<br>For each 12 day streak<br>of daily surveys
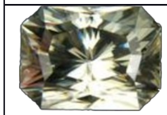	**Agronite**<br>For each 18 day streak<br>of daily surveys
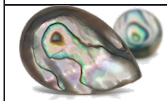	**Shell stone**<br>For each 30 day streak<br>of daily surveys

Rewards for active task streaks

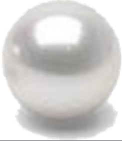	**Japanese Akaya white pearl**<br>For each 3 day streak<br>of active tasks
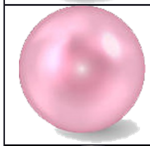	**Pink pearl**<br>For each 6 day streak<br>of active tasks
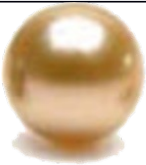	**Southsea golden pearl**<br>For each 12 day streak<br>of active tasks
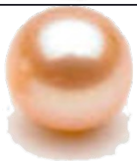	**Orange pearl**<br>For each 18 day streak<br>of active tasks

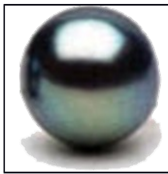	**Taihitian black pearl**<br>For each 30 day streak<br>of active tasks

Rewards for weekly survey streaks

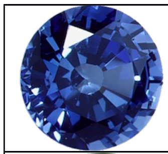	**Saphire stone**<br>After first weekly survey
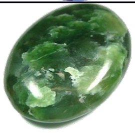	**Jade stone**<br>After 2nd weekly survey
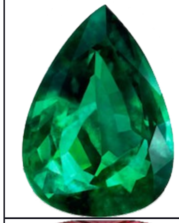	**Emrald**<br>After 3rd weekly survey
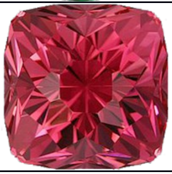	**Red beryl**<br>After 4th weekly survey
