# Supplementary material for: Toward Increasing Engagement in Substance Use Data Collection: Development of the Substance Abuse Research Assistant App and Protocol for a Microrandomized Trial Using Adolescents and Emerging Adults
Source: JMIR Res Protoc. 2018 Jul 18;7(7):e166. doi: 10.2196/resprot.9850 (PMC6070723; doi:10.2196/resprot.9850)
Supplement: Multimedia Appendix 4 [file resprot_v7i7e166_app4.pdf]

Life insights

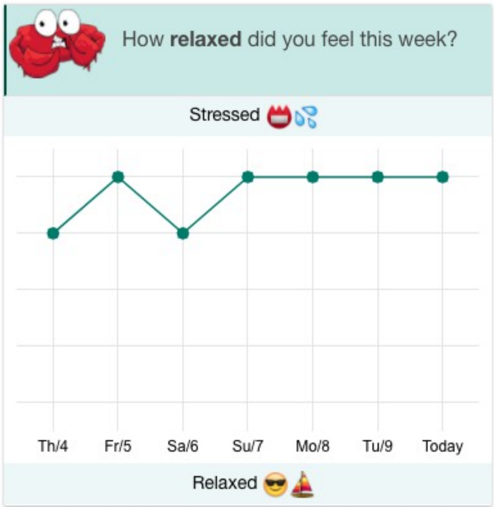

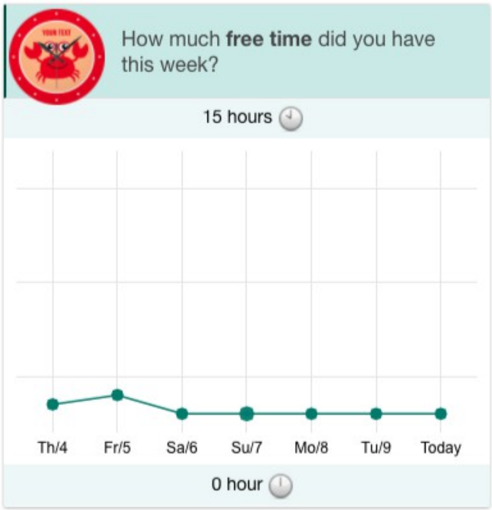

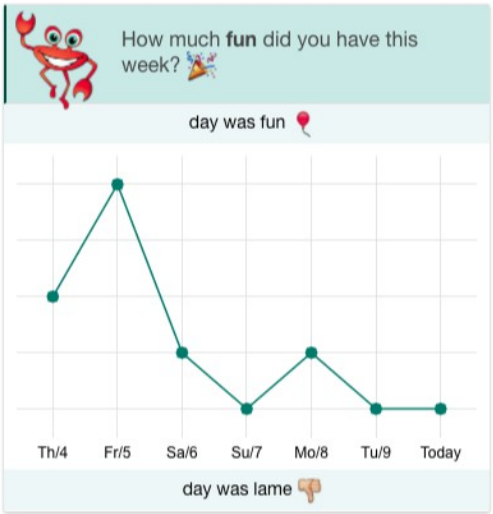

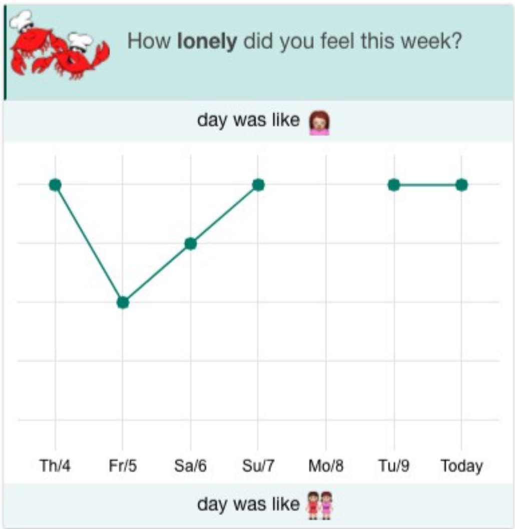
How **lonely** did you feel this week?
day was like
Th/4
Fr/5
Sa/6
Su/7
Mo/8
Tu/9
Today
day was like

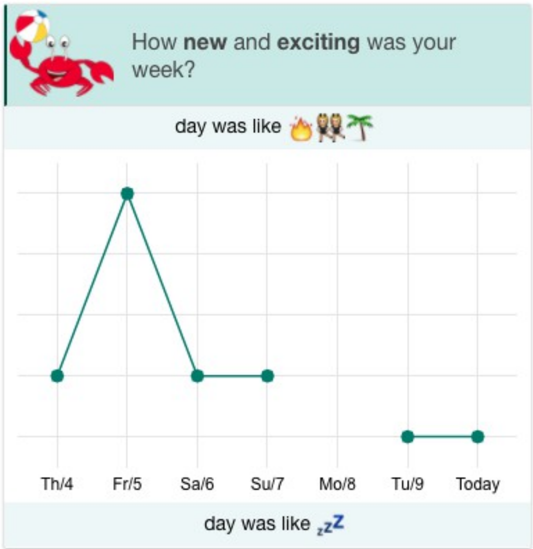
How **new** and **exciting** was your week?
day was like
Th/4
Fr/5
Sa/6
Su/7
Mo/8
Tu/9
Today
day was like

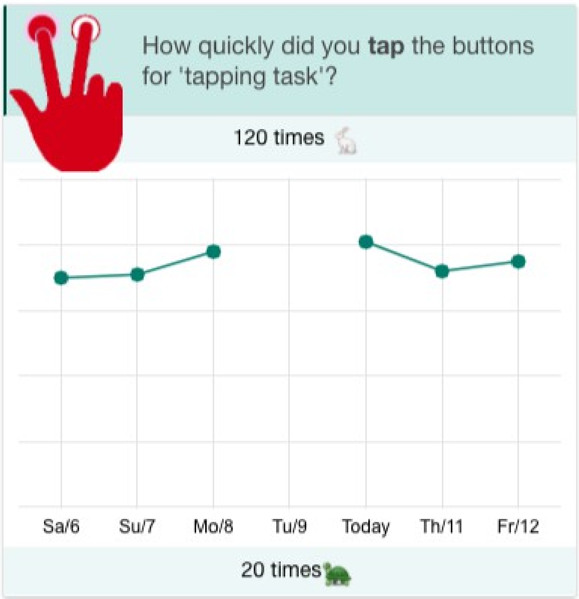
How quickly did you **tap** the buttons for 'tapping task'?
120 times
Sa/6
Su/7
Mo/8
Tu/9
Today
Th/11
Fr/12
20 times

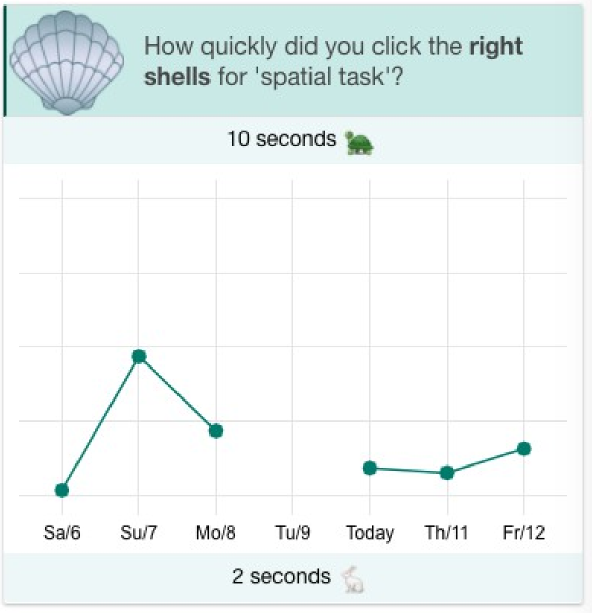
How quickly did you click the **right shells** for 'spatial task'?
10 seconds
Sa/6
Su/7
Mo/8
Tu/9
Today
Th/11
Fr/12
2 seconds
