# Supplementary material for: Toward Increasing Engagement in Substance Use Data Collection: Development of the Substance Abuse Research Assistant App and Protocol for a Microrandomized Trial Using Adolescents and Emerging Adults
Source: JMIR Res Protoc. 2018 Jul 18;7(7):e166. doi: 10.2196/resprot.9850 (PMC6070723; doi:10.2196/resprot.9850)
Supplement: Multimedia Appendix 5 [file resprot_v7i7e166_app5.pdf]

1. "Reach for the stars so if you fall you land on a cloud." - ***Kanye West***
2. "No you’re not perfect but you’re not your mistakes." - ***Kanye West***
3. "You have every right to a beautiful life." - ***Selena Gomez***
4. "I'ma keep running 'cause a winner don't quit on themselves." -***Beyonce***
5. "The world will see you the way you see you, and treat you the way you treat yourself." -***Beyonce***
6. "If you have no shadows then you're not standing in the light." -***Lady Gaga***
7. "You have to be unique and different to shine in your own way." -***Lady Gaga***
8. "Who I wanna be still seems so far away but I know I'm gonna get there someday." ***-Dierks Bentley***
9. "I put my armor on, show you how strong how I am. I put my armor on, I'll show you that I am unstoppable." ***-Sia***
10. "I'm like a rubberband until you pull too hard. I may snap and I move fast but you won't see me fall apart." ***-Sia***
11. "Just really, really believe in what you're trying to do. Don't let people alter that. Let people advise you and lead you down paths to make smart decisions." ***-Luke Bryan***
12. "Build your own pyramids, write your own hieroglyph." ***-Kendrick Lamar***
13. "We all get distracted, the question is, would you bounce back or bounce backwards?" ***-Kendrick Lamar***
14. "I don't wanna be Jordan, I don't wanna be Bird or Isiah, I don't wanna be any of those guys. I want to look in the mirror and say I did it my way." ***-Allen Iverson***
15. "I never worry about the problem. I worry about the solution." ***-Shaquille O'Neal***
16. "I've had to learn to fight all my life - got to learn to keep smiling. If you smile things will work out." ***-Serena Williams***
17. "Even though you're fed up, you gotta keep your head up." ***-Tupac Shakur***
18. "Imma be what I set out to be, without a doubt undoubtedly." ***-Eminem***
19. "The first step is you have to say that you can." ***-Will Smith***
20. "The successful warrior is the average man, with laser-like focus." ***-Bruce Lee***
21. "Sometimes it's the journey that teaches you a lot about your destination." ***-Drake***
22. "Our greatest glory is not in falling but in rising every time we fall." ***-Sylvester***

Stallone

23. "Being different from everyone else just made me a lot stronger." ***-Drake***

24. "You know what they say. Fool me once, strike one, but fool me twice…strike three." ***-Michael Scott***

25. "Being happy is the goal, but greatness is my mission." ***-Childish Gambino***

26. "The motivation for me was them telling me what I couldn't be." ***-Jay Z***

27. "I'm hungry for knowledge. The whole thing is to learn every day. To get brighter and brighter. That's what this world is about." ***-Jay Z***

28. "I seen a baby cry, seconds later he laughs. The beauty of life's pain, pain never lasts." ***-J. Cole***

29. "My mistakes are my biggest professors and learning life lessons." ***-Big Sean***

30. "Dreams stopped being dreams when I turned 'em into goals." ***-Big Sean***

31. "Cherish these nights, cherish these people. Life is a movie, but there'll never be a sequel." ***-Nicki Minaj***

32. "Life is too short to live the same day twice. So each new day, make sure you live your life." ***-Machine Gun Kelly***

33. "Why do we try so hard to fit in, when we were born to stand out?" ***-Machine Gun Kelly***

34. "Life without dreaming is a life without meaning." ***-Wale***

35. "Let your dreams stay big and your worries stay small." ***-Rascal Flatts***

36. "I hope you never look back but you never forget, all the ones who loved you and the place you left." ***-Rascal Flatts***

37. "Don't believe in kings, believe in the Kingdom." ***-Chance the Rapper***

38. "Everybody's somebody's everything." ***-Chance the Rapper***
