# Supplementary material for: Toward Increasing Engagement in Substance Use Data Collection: Development of the Substance Abuse Research Assistant App and Protocol for a Microrandomized Trial Using Adolescents and Emerging Adults
Source: JMIR Res Protoc. 2018 Jul 18;7(7):e166. doi: 10.2196/resprot.9850 (PMC6070723; doi:10.2196/resprot.9850)
Supplement: Multimedia Appendix 6 [file resprot_v7i7e166_app6.pdf]

Protocol for Texting Non-Compliant Participants during the Field Testing Period

Impetus for protocol: When participants begin to disengage from completing their daily surveys and active tasks, we want to have a systematic way of sending them reminders without cancelling out the signal of our engagement strategies in the app**.** To accomplish this, the following steps will be taken to remind non-compliant participants about completing their daily data collection.

When a participant misses a survey or active task…

1. **The Next Day**: A staff member will text the participant between 9pm and 10pm using our IRB-approved message template. This contact will be recorded in our contact notes Access database.
2. **On Day 5**: If the participant is still non-compliant and has not answered *any* surveys or active tasks, a staff member will text the participant again between 9pm and 10pm using our IRB-approved message template. This contact will be recorded in our contact notes Access database.
3. **After Day 7:** If the participant is still non-compliant and has not answered any surveys or active tasks, staff will call the participant between Day 8 – Day 10 at their preferred time of day (morning, afternoon or evening) to see if their app is working OK. This contact will be recorded in our contact notes Access database.
4. **Three days after the reminder call is made:** If the participant is still non-compliant, we will start with step 1 again and text message the participant between 9pm and 10pm using our IRB-approved message template. This contact will be recorded in our contact notes Access database.

These three steps will be repeated until either the participant resumes completing their surveys/active tasks or we've followed up with the participant a total of 9 times (per our IRB protocol). Once a participant has been contacted a total of 9 times, we will not contact them again until it's time to remind them about their follow-up appointment.

Some participants will have unique circumstances that merit exceptions to this protocol. These exceptions include:

1. If a participant uninstalls the app: Staff will call the participant within 3 days of the app being uninstalled to find out why the app was uninstalled and ask if they would be willing to reinstall the app. This contact will be recorded in our contact notes Access database.
2. If a participant is not able to download the app during their intake visit: If the participant's username does not appear on the adherence website 2 days after their intake visit, staff will call the participant to see how the app is working and ask if they have any

questions about how to use it. This contact will be recorded in our contact notes Access database.

3. <u>If a participant does not have voice and/or text messaging capabilities</u>: Staff will contact the participant by their preferred method of contact (email, social media message) and use IRB-approved email/social media templates in place of text message reminders. In place of a reminder call every 7 days, staff will send a personalized email/social media message. These contacts will be recorded in our contact notes Access database.

As new exceptions emerge, Meredith and Maureen will bring these cases to the next PI meeting so these exceptions can be discussed by the team on a case-by-case basis.

Text messaging template

<u>If participant has missed one survey/active task</u>

Hey _________! It's SARA! You missed a survey/active tasks recently. Is your app working OK? Remember that you can unlock fish and sometimes a money bonus for completing your survey and active tasks each night between 6pm and 12am. Questions? Reply or email us at sarasquad@med.umich.edu! Thx!

<u>If participant has missed several surveys/active tasks</u>

Hey _________! It's SARA! You missed a few surveys/active tasks recently. Is your app working OK? Remember that you can unlock fish and sometimes a money bonus for completing your survey and active tasks each night between 6pm and 12am. Questions? Reply or email us at sarasquad@med.umich.edu! Thx!

<u>Initial FU reminder</u>

Hey ______! It's SARA! Your follow-up phone appointment is [day, date] at _:__ [am/pm]. We'll call you at this number for the appointment. For doing it, you'll receive a $30 ______ gift card. Does this appointment still work for you? Reply Y=Yes or N=No (and we'll contact u soon to reschedule). Thx!
