# Supplementary material for: Toward Increasing Engagement in Substance Use Data Collection: Development of the Substance Abuse Research Assistant App and Protocol for a Microrandomized Trial Using Adolescents and Emerging Adults
Source: JMIR Res Protoc. 2018 Jul 18;7(7):e166. doi: 10.2196/resprot.9850 (PMC6070723; doi:10.2196/resprot.9850)
Supplement: Multimedia Appendix 7 [file resprot_v7i7e166_app7.pdf]

1: What do you dislike, for no good reason?

- Mayonnaise
- Dryer lint
- People who brag on Facebook
- The word “moist”
- Name brand addicts
- Country music
- Reality TV
- Other

2: If you were stranded on a desert island, what one food, what would you bring?

- Ramen Noodles
- Cookie dough ice cream
- Nacho cheese Dorito’s
- Power bars
- Pop Rocks
- French fries
- Forget food, I’m bringing coffee
- Other

3: What movie best describes your life?

- Breakfast Club
- A Nightmare on Elm Street
- Love Actually
- Clueless
- Kickass
- Diary of a Wimpy Kid
- Barely Legal
- Other

4: If you were an animal, what would you be?

- Dinosaur
- Alligator
- Unicorn
- Cockroach
- Wallaby
- Dragon
- Charizard
- Other

5: If you could hang out with a movie character for one day, who would you pick?

- Harry Potter
- Ironman
- Jack Sparrow from Pirates of the Caribbean
- Fat Amy from Pitch Perfect
- Katniss Everdeen from Hunger Games
- Napoleon Dynamite
- Elle Woods from Legally Blonde

Other
