# Supplementary material for: Toward Increasing Engagement in Substance Use Data Collection: Development of the Substance Abuse Research Assistant App and Protocol for a Microrandomized Trial Using Adolescents and Emerging Adults
Source: JMIR Res Protoc. 2018 Jul 18;7(7):e166. doi: 10.2196/resprot.9850 (PMC6070723; doi:10.2196/resprot.9850)
Supplement: Multimedia Appendix 8 [file resprot_v7i7e166_app8.pdf]

Primary Analysis of SARA

The primary analysis for the two hypotheses mentioned in the "Inference criteria" section is as follows:

1 Hypothesis 1: 4PM push notification

1.1 Overview of hypothesis

H_1: The 4PM push notification with inspirational quote will increase the full completion of survey and/or active task the same day as compared to no inspirational quote ($p < 0.025$)

H_0: The 4PM push notification with inspirational quote will *not* increase the full completion of survey and/or active task the same day as compared to no inspirational quote

- Primary outcome: participants fully complete the survey and/or active tasks in the evening of the same day. This outcome is binary.
- Independent variable: push notification with an inspirational message vs no push notification with an inspirational message. This variable is binary.
- Covariates: whether the survey and/or active tasks were fully completed prior-day (binary); whether text or phone calls were made in the last 24 hours, i.e. after 4PM from the previous day to before 4PM the current day (binary); whether the app was opened in last the prior 72 hours outside of when survey and/or active task were completed, i.e. after 4PM from the 3-days ago to before 4 PM the current day (binary)

1.2 Notation

Denote the primary outcome for person i on day t ($t = 1, ..., 30$ and $i = 1, ..., n$ where n is the total sample size.) by Y_{it}. There are 3 binary covariates in both of the above analyses. Denote the 3 by 1 covariate vector for the ith person on the tth day by X_{it}. Denote the binary indicator of treatment for the ith person on the tth day by A_{it}.

1.3 Construction of the Test Statistic

We will use a multiplicative structural nested log linear model for a binary outcome. To conduct the hypothesis tests we estimate the marginal effect of treatment on the log linear scale. The marginalization is over time and the individual's data, H_{it}. Technically this marginal effect is given by:

$$\log\left(\frac{\sum_{t=1}^{30} E[Y_{it}=1|A_{it}=1]}{\sum_{t=1}^{30} E[Y_{it}=1|A_{it}=0]}\right)=\beta_0.$$

β_0 is a regression coefficient in a multiplicative structural nested log-linear mean model [1, 2].

We use an analysis method that permits us to include covariates, X_{it} to reduce the noise. In particular we use the working model:

$$e^{X_{it}^T\alpha_0}\approx E[e^{-A_{it}\beta_0}Y_{it}|X_{it}].$$

This model need not be correct for the estimator of β_0 to be consistent. This model is used to reduce estimation variance.

We estimate the marginal treatment effect by solving

$$0=\sum_{i=1}^{n}\sum_{t=1}^{30} e^{-A_{it}\hat{\beta}}\left(Y_{it}-e^{X_{it}^T\hat{\alpha}+A_{it}\hat{\beta}}\right)\begin{pmatrix}(A_{it}-1/2)\\ -e^{X_{it}^T\hat{\alpha}}X_{it}\end{pmatrix} \quad (1)$$

to obtain $\hat{\beta}$ and $\hat{\alpha}$.

The approximate variance-covariance matrix

$$Var\begin{pmatrix}\sqrt{n}(\hat{\beta}-\beta_0)\\ \sqrt{n}(\hat{\alpha}-\alpha_0)\end{pmatrix}=M_n^{-1}\Sigma_n\left(M_n^{-1}\right)^T$$

where

$$M_n=(1/n)\sum_{i=1}^{n}\sum_{t=1}^{30}\begin{pmatrix}(A_{it}-1/2)e^{-A_{it}\hat{\beta}}Y_{it}A_{it} & (A_{it}-1/2)e^{X_{it}^T\hat{\alpha}}X_{it}^T\\ e^{-A_{it}\hat{\beta}}Y_{it}A_{it}e^{X_{it}^T\hat{\alpha}}X_{it} & e^{X_{it}^T\hat{\alpha}}X_{it}X_{it}^T\end{pmatrix}$$

and

$$\Sigma_n=(1/n)\sum_{i=1}^{n}\left(\sum_{t=1}^{30}\left(e^{-A_{it}\hat{\beta}}Y_{it}-e^{X_{it}^T\hat{\alpha}}\right)\begin{pmatrix}(A_{it}-1/2)\\ e^{X_{it}^T\hat{\alpha}}X_{it}\end{pmatrix}\right)*\left(\sum_{t=1}^{30}\left(e^{-A_{it}\hat{\beta}}Y_{it}-e^{X_{it}^T\hat{\alpha}}\right)\begin{pmatrix}(A_{it}-1/2)\\ e^{X_{it}^T\hat{\alpha}}X_{it}\end{pmatrix}\right)^T$$

1.4 Test Statistic

For the test $H_0=\beta_0\le 0$ versus $H_1=\beta_0>0$, we reject H_0 in favor of H_1 if $T_n>z_{.025}$ where $z_{.025}$ is the 97.5th percentile of the standard normal distribution and $T_n=\frac{\sqrt{n}\hat{\beta}}{\sigma_n}$ and σ_n is the square root of the $(1,1)$ entry in $M_n^{-1}\Sigma_n\left(M_n^{-1}\right)^T$.

2 Hypothesis 2: After-survey-completion reward

2.1 Overview of hypothesis

H_1: Among individuals who complete the survey, offering a post-survey-completion meme will increase the full completion of survey and/or action task the next day as compared to not offering a meme after survey completion ($p < 0.025$)

H_0: Among individuals who complete the survey, offering a post-survey-completion meme will *not* increase the full completion of survey and/or action task the next day as compared to not offering a meme after survey completion

- Primary outcome: whether participants fully complete the survey and/or active tasks the following day. This outcome is binary
- Independent variable: offering a meme vs. not offering a meme after survey completion. This variable is binary.
- Covariates: whether the survey and/or active tasks were fully completed the prior day (binary); whether text or phone calls were made in the last 30 hours (binary); whether the app was opened in the prior last 80 hours outside of when survey and/or active task were completed, i.e. after 6PM from the 3-days ago to before data collection time on the current day (binary)

2.2 Notation

Denote the primary outcome for person i on day t ($t = 1, ..., 29$ and $i = 1, ..., n$ where n is the total sample size.) by $Y_{i(t+1)}$. There are 3 binary covariates in both of the above analyses. Denote the 3 by 1 covariate vector for the ith person on the tth day by X_{it}. Denote the binary indicator of treatment for the ith person on the tth day by A_{it}. In this second analysis, person i is only available for treatment on day t if this person fully completed the survey. Let $I_{it} = 1$ if person i completed the survey on day t and set $I_{it} = 0$ otherwise. Note that in the first analysis $I_{it} = 1$ for all i,t.

2.3 Construction of the Test Statistic

We will use a multiplicative structural nested log linear model for a binary outcome. To conduct the hypothesis tests we estimate the marginal effect of treatment on the log linear scale. The marginalization is over time and the individual's data, H_{it}. Technically this marginal effect is given by:

$$\log\left(\frac{\sum_{t=1}^{29} E[Y_{i(t+1)} = 1 | A_{it} = 1, I_{it} = 1]}{\sum_{t=1}^{29} E[Y_{i(t+1)} = 1 | A_{it} = 0, I_{it} = 1]}\right) = \beta_0.$$

β_0 is a regression coefficient in a multiplicative structural nested log-linear mean model [1, 2].

We use an analysis method that permits us to include covariates, X_{it} to reduce the noise. In particular we use the working model:

$$e^{X_{it}^T \alpha_0} \approx E[e^{-A_{it}\beta_0} Y_{i(t+1)} | X_{it}, I_{it} = 1].$$

This model need not be correct for the estimator of β_0 to be consistent. This model is used to reduce estimation variance.

We estimate the marginal treatment effect by solving

$$0 = \sum_{i=1}^{n} \sum_{t=1}^{29} I_{it} e^{-A_{it}\hat{\beta}} \left(Y_{i(t+1)} - e^{X_{it}^T \hat{\alpha} + A_{it}\hat{\beta}} \right) \begin{pmatrix} (A_{it} - 1/2) \\ -e^{X_{it}^T \hat{\alpha}} X_{it} \end{pmatrix} \tag{2}$$

to obtain $\hat{\beta}$ and $\hat{\alpha}$.

The approximate variance-covariance matrix

$$Var \begin{pmatrix} \sqrt{n}(\hat{\beta} - \beta_0) \\ \sqrt{n}(\hat{\alpha} - \alpha_0) \end{pmatrix} = M_n^{-1} \Sigma_n \left(M_n^{-1} \right)^T$$

where

$$M_n = (1/n) \sum_{i=1}^{n} \sum_{t=1}^{29} I_{it} \begin{pmatrix} (A_{it} - 1/2) e^{-A_{it}\hat{\beta}} Y_{i(t+1)} A_{it} & (A_{it} - 1/2) e^{X_{it}^T \hat{\alpha}} X_{it}^T \\ e^{-A_{it}\hat{\beta}} Y_{i(t+1)} A_{it} e^{X_{it}^T \hat{\alpha}} X_{it} & e^{X_{it}^T \hat{\alpha}} X_{it} X_{it}^T \end{pmatrix}$$

and

$$\Sigma_n = (1/n) \sum_{i=1}^{n} \left(\sum_{t=1}^{29} I_{it} \left(e^{-A_{it}\hat{\beta}} Y_{i(t+1)} - e^{X_{it}^T \hat{\alpha}} \right) \begin{pmatrix} (A_{it} - 1/2) \\ e^{X_{it}^T \hat{\alpha}} X_{it} \end{pmatrix} \right) * \left(\sum_{t=1}^{29} I_{it} \left(e^{-A_{it}\hat{\beta}} Y_{i(t+1)} - e^{X_{it}^T \hat{\alpha}} \right) \begin{pmatrix} (A_{it} - 1/2) \\ e^{X_{it}^T \hat{\alpha}} X_{it} \end{pmatrix} \right)^T$$

2.4 Test Statistic

For the test $H_0 = \beta_0 \leq 0$ versus $H_1 = \beta_0 > 0$, we reject H_0 in favor of H_1 if $T_n > z_{.025}$ where $z_{.025}$ is the 97.5th percentile of the standard normal distribution and $T_n = \frac{\sqrt{n}\hat{\beta}}{\sigma_n}$ and σ_n is the square root of the $(1, 1)$ entry in $M_n^{-1} \Sigma_n \left(M_n^{-1} \right)^T$.

References

[1] James M Robins. Correcting for non-compliance in randomized trials using structural nested mean models. *Communications in Statistics-Theory and methods*, 23(8):2379–2412, 1994.

[2] James M Robins. Causal inference from complex longitudinal data. *Latent variable modeling and applications to causality*, pages 69–117, 1997.
